# Favus

**Published:** 1886-10

**Authors:** Henry J. Reynolds

**Affiliations:** Professor of Dermatology, College of Physicians and Surgeons, Chicago


### FAVUS—A CLINICAL LECTURE.

*By* HENRY J. REYNOLDS, M. D., *Professor of Dermatology, College of Physicians and Surgeons, Chicago.*

[Reported by B. VAN SWERINGEN.]

GENTLEMEN:

I wish today to call your attention to the study of a disease which belongs to a different class from those which we have recently been considering. It belongs to the class known as "Parasitic Diseases" of the skin. We have, as most of you are aware, two principal forms of parasite giving rise to the diseases of this class, viz.: "animal" and "vegetable." The various forms of pediculosis and scabies constitute the diseases caused by the animal parasite. Arising from vegetable origin we have three forms of disease, the manifestations of each being somewhat different, as likewise, the vegetable parasite, fungus or growth giving rise to them. They are "tinea versicolor" (a case of which you saw a few days ago), "tinea trichophytina," or ringworm (under which head three different manifestations, differing only on account of location, are embraced, viz.: tinea cincinata, or ringworm of the non-hairy skin; tinea barbae, tinea sycosis, or barbers' itch, which is the same thing, as it occurs in the bearded region, and tinea tonsurans, which is also the same thing only occurring in the scalp), and "tinea favosa," or "favus." It is to the last named disease that I will now confine my remarks.

Before examining the two patients before you, which are both typical cases of favus, let me ask that you first get a good general idea of the symptoms, characteristics, and clinical history of the disease, without which it would be impossible to examine and study the disease, as it manifests itself in these or any other cases, intelligently.

Being a parasitic disease, it of course results from contagion; its contagious character may then be mentioned as the first characteristic of the disease.

It is always a scalp disease, though the non-hairy skin, and even the nails, may occasionally be coincidently affected, as in the plate I now show you.

It is a comparatively rare disease, occurring with about the same frequency as lupus erythematosus, and, like that disease, is much more common in Europe than in this country.

The disease is first seen as one or several small yellow spots beneath or within the epidermis.

In from two to four weeks these spots enlarge and form pea-sized, cup-shaped, yellow, sulphur-colored crusts, each penetrated by one or more hairs.

These cup-shaped crusts, now, upon removal, leave a correspondingly depressed, moist, reddened surface.

As the disease advances these lesions, which are as yet discrete, become confluent and fused into a general, continuous mass of yellow, sulphur-colored crusting; though here and there, especially near the margins the cup-shaped character may usually be observed.

The condition, as I now describe it, represents the disease as usually first seen by the physician, and may, if of very long standing, involve almost the entire scalp, or may be confined to patches here and there.

The crusts in this disease always have their own peculiar consistence, being more friable and brittle, perhaps, than the crusts of any other form of skin disease, breaking down almost like cheese.

There is usually not much inflammatory trouble, though the skin is usually hyperaemic and pus may be formed: the crusting, however, as demonstrated by the microscope, is largely composed of the fungous growth rather than of dried pus or other inflammatory product.

The hairs, including the follicles, being invaded by the parasitic growth, now become loose, lustreless and brittle.

If the disease continue, the follicles, from the irritation and inflammatory action, become in time completely destroyed and the hairs are not reproduced, leaving smooth, partly bare places sometimes, here and there, as a relic of the disease, it having then undergone, to some extent, spontaneous cure in such places.

The disease may be limited to one or two small patches or it may involve almost the entire scalp, though there is no tendency to symmetrical distribution as in seborrhœa, psoriasis, etc.

It is a very chronic affection, lasting sometimes for a great many years.

It generally begins in childhood.

The subjective sensation is usually that of mild or moderate itching.

The crusts may be all removed by careful washing and cleansing, leaving a surface apparently almost free from disease, but under favorable circumstances—heat and moisture—they will be very rapidly reproduced; giving in a very few weeks the same appearance as before.

The affected part has a characteristic odor, described as resembling the smell of mice.

The disease may, lastly, be characterized by its transmissibility to or from numerous lower animals, as cats, dogs, rabbits, mice, etc.

The disease is of course not a constitutional one, but purely local.

CASE I.—The history of this boy's case is substantially as follows: He is eleven years of age, and has had the disease for seven years. Otherwise he is apparently healthy. He gives us no history of contagion as a cause of the disease in his case, but upon questioning him we find that a little brother has a similar spot on his scalp, which would tend to show the first characteristic referred to, viz., contagion.

CASE II.—In the history of this case there is nothing of particular interest, more than that he is twenty-five years of age, and has been afflicted with the disease for seventeen years. He, like the boy, has been treated more or less by numerous physicians during the entire career of the disease, without receiving any permanent benefit; this fact tending to show the very stubborn and chronic character of the disease.

The patients will now pass around among you, and you will please observe the various points which I have enumerated as characteristic of favus. You will first readily observe that the disease is confined to the scalp; but the earlier manifestations of the disease you will be unable to see in these cases of so long standing. Less than two weeks ago the crusts, by treatment, were almost entirely removed, but, in order that you might see and study the disease in its typical, untreated state, I have allowed them to re-accumulate undisturbed. Toward the margin, where the lesions are more or less discrete, may be seen some of the characteristic cup-shaped crusts. You will observe that the crusting is yellowish or sulphur-colored, that it is very brittle, differing in the latter respect from crusting composed of a purely inflammatory product; and that when the crust is removed we do not find the active inflammatory condition of skin characteristic of truly inflammatory diseases, as eczema, for instance. I will next call your attention to the condition of the hairs in these cases. You will observe that they are dry and lustreless, loose, brittle, and broken off. You will observe in Case II a partly bald, smooth patch. This represents a place where the disease has undergone spontaneous cure, the follicles having become so involved in the process that the hairs have fallen out and were not reproduced. You will find, if you direct your attention to the odor of the affected parts, that you get the characteristic so-called smell of mice. We have stated that children were more susceptible to the disease, and we find in both of these cases that the disease was contracted in childhood. As regards subjective sensation, we find that while there is some itching in these cases it is of a very mild or insignificant character. Thus we find in these cases most of the symptoms referred to as characteristic of favus.

*Etiology.*—As the disease is a parasitic one, it is like all the other diseases of this class, whether of animal or vegetable origin, caused by the development of a parasite. The parasite in this, as in all the vegetable parasitic diseases, is a vegetable organism, fungus or growth, and is received and communicated by contact; the disease being therefore a contagious one. As previously stated, the disease is met with in certain of the lower animals, and may therefore be derived from this source. It is likewise, of course, communicable from one person to another, the only requisite being a favorable soil for the development of the fungus when deposited by contact; the most favorable site being the scalp. The disease may be communicated through the medium of certain articles upon which some of the parasitic growth has accidentally or otherwise been deposited, as articles of clothing, towels, combs and brushes, etc. Like all other diseases, some individuals are more susceptible to it than others. It is usually met with among children, especially the uncleanly; adults being peculiarly exempt from it.

*Pathology.*—The microscopical organisms or vegetable growth to which the disease is due, upon being deposited upon the skin, gains lodgment beneath some of the superficial layers of the epidermis, particularly in the depression around the roots of the hairs. It then, as it grows and multiplies, burrows in all directions, down into the follicles, into the hair bulbs, and even the tissues of the shaft of the hair for a short distance from the scalp, though not to the same extent as the similar parasite of tinea tonsurans. They soon become so aggregated as to form a small yellow spot with a thin covering in the horny layer. This soon increases in size and forms the characteristic yellow cup-shaped crust, and is, of course, composed almost entirely of the vegetable growth. The nutrition of the hair is thus interfered with and in due time the hair loses its lustre, becomes brittle, the bulb atrophies, loosens, and the hair falls out ; it may grow in again, but if the disease continue long enough the follicle becomes destroyed and the hair is not reproduced and the partial baldness referred to is thus brought about.

In the epidermis the parasite works very superficially, but the deep or true skin, though not invaded by the growth, is in an irritable, somewhat inflammatory condition. Further reference will be made to the character of the parasite at the close of the lecture, when you may have an opportunity of studying it under the microscope.

*Diagnosis.*—The recognition of a typical case of favus, or in other words a case wherein most of the symptoms and characteristics just considered are present, is a matter of no great difficulty, providing we know and are familiar with what those characteristics of favus are. The disease is not always typical, however ; for instance, if the patient when first seen has been under treatment or has given the scalp ordinary good care in the way of washing, etc., the crusts may be moderate or even absent, and it would then be very difficult to make a diagnosis from the appearance of the part. In this case, the history given by the patient of the previous character and behavior of the scap, as regards crusting, the color of the crusts, etc., would arouse suspicions of favus. In any event, the use of the microscope would settle the question. The affections presenting the most striking resemblance are eczema, tinea tonsurans or ringworm of the scalp, and psoriasis.

We have the parasitic or contagious origin in favus. In eczema, though many may have it at the same time, it is not propagated from one person to another. We have much greater irritation, more serous exudation and itching in eczema than in favus. The crusts, though sometimes inclined to be yellow in eczema, never have the bright yellow of favus, but rather a dirty yellow. The crusts in eczema never have the brittle quality that is characteristic of favus; they never have the umbilicated condition. In eczema the disease is not so liable to be continuously confined to the same patch as in favus, but will usually be more generalized and quite probably show an irritated, red, eczematous condition in the face at some time during the career of the disease. The hairs in eczema do not show the brittle, lustreless quality, with a tendency to fall out, that is characteristic of favus. As to the odor of the part, there may be a very offensive odor in eczema under certain conditions, but it is of an entirely different character from that previously referred to as characteristic of favus. When uncertainty exists, or in any event, the diagnosis can always be very readily made by the use of the microscope, to which we will refer at the close of the lecture.

In tinea tonsurans we do not have the yellow crusts, in fact we do not have crusts at all, but scales. The disease spreads centrifugally by peripheral extension, etc.

I have seen psoriasis of the scalp diagnosed as favus. In this disease, though the scales may at times accumulate into a crust-like mass with a dirty yellowish color, there need rarely be much trouble in discriminating one from the other. Even without the microscope the scaly or crusty accumulations of psoriasis never have the sulphur-yellow color, they are never cup-shaped, they are more general, almost always involving various portions of the non-hairy integument. The hair retains its normal lustre, is not brittle, broken off and loose, etc. If psoriasis have lasted for years, it will generally give a history of having been, at some time during its career, entirely well, which is never the case in favus.

*Treatment.*—The disease being dependent upon a parasitic or fungous growth, it naturally follows that remedies which destroy the growth will cure the disease, providing they be applied so as to reach it; but this is the great and only difficulty in treating favus. The parasitic growth being a very small, microscopical one, it penetrates to the bottom of the hair-follicles and even into the hair structure itself, and is therefore very difficult to reach with the remedy. The class of remedies to be used is that known as parasiticides, or remedies capable of destroying vegetable organisms, and it matters not which of the long list be used, though it is probable that certain combinations penetrate to the deeper parts more readily than others and would therefore be preferable.

It is necessary first, as in treating every other diseased surface, to remove the crusting, which can be very readily accomplished by frequent anointing and washing with soap and hot water. This being accomplished, we know now that if the hairs be removed—which are usually loose in the diseased patch—every place invaded by the growth can readily be reached by the local application. This procedure is undoubtedly not always necessary, but in stubborn, old standing cases, like those before you, it will be found a more satisfactory way of treating the disease. In this way the cure is certain, while to treat without removing the hairs is always tedious and uncertain. Whether epilation be resorted to or not, the remedy must be thoroughly rubbed in day after day for a month or two at a time. It is well then to discontinue for a week or so to see if the crusting be reproduced, and to what extent, and if any disease be found remaining the process must be again repeated in like manner till the crusts cease to reappear.

In these cases we will order that the hair be shingled and that the parts be anointed with some oleaginous preparation —of which there is nothing better than lard—three times a day, and washed with soap and hot water once a day. Then in a few days, as soon as well freed from crusts, we will have the hairs removed. This should be done by the physician or a competent assistant. We will then apply three times a day a solution of ten grains of bichloride of mercury in an ounce of chloroform. This we will continue as previously suggested for a month or so, etc.

*Prognosis.*—The prognosis in a case treated in this way is always good, providing the treatment be rigidly and carefully persisted in for a sufficient length of time, which may be many months.

Let us now study briefly this vegetable parasite, or fungous growth under the microscope, by which means we are enabled not only to arrive at a positive diagnosis, but to demonstrate what we have said of the pathology of the disease. I now take, as you see, a very small piece of the crust from this patient's head, moisten it on the slide with a little water, put a cover glass over it, and place it under a one-sixth objective. The fungus can now be seen in great abundance. You will now notice first, as you examine the specimen, small round, globular or oblong bodies. You may also see small elongated tubular bodies, some of which are chain-like in appearance, of a similar diameter. The small round bodies which you see in great numbers are what are termed spores; they are not exactly uniform in size, and vary from about $\frac{1}{7000}$ to $\frac{1}{9000}$ of an inch in diameter, showing therefore, as you can see, about one-third to one-half the diameter of a red blood corpuscle as seen under the same power. The tubular, thread-like elongations you see are termed mycelia, and the chain-like bodies are either spores linked together, or mycelia traversed by transverse septa, which in all probability ultimately divide into spores.

